# The taxonomic, functional and phylogenetic diversity of birds in Xiaohongxiang Wetland, southwest China

**DOI:** 10.3897/BDJ.12.e136248

**Published:** 2024-12-04

**Authors:** Binqiang Li, Shaohui Zhang, Jie Guo, Shanjun Ma, Wenjie Zhang

**Affiliations:** 1 Faculty of Biodiversity and Conservation, Southwest Forestry University, Kunming, China Faculty of Biodiversity and Conservation, Southwest Forestry University Kunming China; 2 Planing and Design Institute, Yunnan Forestry Technological College, Kunming, China Planing and Design Institute, Yunnan Forestry Technological College Kunming China

**Keywords:** wetland ecosystems, bird diversity, community assembly, Xiaohongxiang

## Abstract

Small wetlands are essential for preserving global biodiversity, yet they are frequently neglected in conservation strategies due to vague definitions and a lack of research attention. In this study, we conducted thirty-six surveys via the point count method in the Xiaohongxiang Wetland, Anning City, Yunnan Province, China, from November 2023 to June 2024. We aimed to evaluate the impact of various habitats surrounding Xiaohongxiang Wetland (wetlands, villages, farmlands, cherry plantations and pine forests) on the taxonomic, functional and phylogenetic diversity of avian species and investigate the significance of these habitats for ecological conservation and restoration efforts. A total of 62 species were recorded. Small wetlands are vital for supporting common waterbirds, but may not safeguard rare species effectively. While cherry plantations and pine forests enhance avian diversity near wetlands, their low functional diversity could limit the ecological niches available to birds. This indicates that monoculture plantations might restrict the habitat variety needed for a diverse bird community. Our study found no clear phylogenetic clustering or overdispersion amongst bird species across habitats, suggesting that community assembly is shaped by competitive exclusion, habitat filtering and neutral processes. Despite a limited sample size, our results highlight a gap between taxonomic and functional diversity, indicating that multidimensional biodiversity metrics are essential for thoroughly evaluating wetland restoration and habitat impacts on bird diversity.

## Introduction

Wetland ecosystems play a crucial role in global ecological conservation by providing a wide range of resources and ecological services to humanity, including water source protection, flood storage and climate regulation (Zedler and Kercher 2005, Davidson et al. 2019). However, human-induced land-use changes and climate change have driven widespread loss and degradation of wetland ecosystems (Sievers et al. 2017, Xiong et al. 2023, Fluet-Chouinard et al. 2023, Shen et al. 2024). For example, between 2003 and 2013, China experienced a notable reduction in its wetland areas, with a total reduction of 3,396,300 ha including a decline of 3,376,200 ha in natural wetlands(Pei et al. 2023). Unfortunately, compared to large wetlands, small wetlands (wetlands smaller than 8 ha) have consistently been neglected in conservation plans, primarily due to inconsistent definitions and a lack of research focus, highlighting an urgent need for more comprehensive studies (Cui et al. 2021, Shen et al. 2024). Small wetlands constitute over 80% of the total wetland area in east Africa; however, their rapid depletion has led to soil salinisation and degradation, undermining agricultural productivity and social stability (Mwita et al. 2013). The degradation and loss of biodiversity in small wetlands have emerged as urgent global environmental issues. Although wetland restoration projects have been implemented worldwide, the effectiveness of these initiatives and the myriad of influencing factors involved are complex and varied (Nakamura et al. 2006, Liu et al. 2024, Bega et al. 2024). Consequently, gaps remain in our understanding of the effectiveness of various restoration methods for enhancing biodiversity.

Numerous regional studies have consistently indicated that crop cultivation, urban development and plantations are the primary causes of natural wetland depletion globally, frequently resulting in variations in the diversity of local species (Johnston 2013, Mao et al. 2018). The fact that changes in wetland land use have taken place globally needs to be acknowledged. The optimisation of ecological services provided by wetlands and their surrounding habitats is one of the primary concerns actively addressed by ecologists and conservationists. Therefore, it is crucial to consider the restoration of surrounding ecosystems in the wetland restoration process. Adjacent ecosystems, such as forests and plantations, can support diverse biodiversity and offer wildlife nesting and shelter opportunities (Ma et al. 2009, Sinha et al. 2019, Qiu et al. 2024). These landscape types play a crucial role in upholding the ecological integrity and functionality of the wetland ecosystem. A deep understanding of the complex connections between wetlands and their surrounding ecosystems is essential for achieving sustainable ecological recovery and ensuring the long-term success of restoration efforts (Liu et al. 2024). However the effectiveness of forests, farmland and plantations around wetlands in enhancing biodiversity remains a subject of debate (Meli et al. 2014, Qiu et al. 2024).

Birds are a vital component of wetland ecosystems; their diversity is not only indicative of the health of ecosystems, but also serves as a critical metric for assessing the effectiveness of wetland restoration efforts (Ma et al. 2009, Luck et al. 2012, Qiu et al. 2024). However, current studies on bird diversity in wetlands primarily focus on taxonomic diversity, with limited emphasis on functional and phylogenetic diversity (Sinha et al. 2019, Luo et al. 2019, Jia et al. 2020, Wang et al. 2021, Rivera et al. 2021, Yuan et al. 2021, Htay et al. 2023, Qiu et al. 2024). The concept of functional diversity (FD) offers insights into how various characteristics of organisms contribute to ecosystem functioning, enhancing our understanding of the complex dynamics (Mason et al. 2005, Laliberté and Legendre 2010, Mouchet et al. 2010). It is crucial as it pertains to the variety of roles that species play in the ecosystem, focusing on their functional rather than their taxonomic identity. It encompasses morphological, physiological and reproductive features that impact species' performance, fitness and roles in the ecosystem. Phylogenetic diversity (PD) refers to the evolutionary relationships and histories amongst species (Faith 1992, Webb 2000, Miller et al. 2018, Owen et al. 2019). Maximising phylogenetic diversity can ensure that a wide variety of forms and functions are present within a species set, which is especially important in species-poor clades or regions or in the absence of meaningful data on functional traits. By comparing bird diversity across various habitats, we can gain insights into the contributions of different habitats to bird diversity and their ecological significance (Devictor et al. 2010, Zhang et al. 2020, Qiu et al. 2024). Additionally, to date, there is limited understanding regarding the impact of cherry plantations on avian biodiversity (Calviño-Cancela 2013, Castaño-Villa et al. 2019).

In 2020, a restoration project was started in the Xiaohongxiang Wetland (hereafter Xiaohongxiang) in Yunnan Province, China. The wetland restoration project primarily includes measures to remove invasive species, plant trees and reduce soil erosion. Human activities, including farmlands and extensive plantations, have significantly impacted the wetlands. Importantly, there is no definitive bird list for Xiaohongxiang. In the present study, we aim to comprehensively understand the influence of different habitats within the Xiaohongxiang on bird taxonomic diversity, functional diversity and phylogenetic diversity by comparing five habitats (wetlands, villages, farmlands, cherry plantations and pine forests) and explore the implications for ecological conservation and restoration efforts. We address the following questions: (ⅰ) Does the restored Xiaohongxiang wetland exhibit a higher diversity of waterbirds, including any endangered or rare species that have found a habitat within the wetland? (ⅱ) To what extent do plantations and human-modified landscapes (such as villages and farmlands) contribute to bird diversity in comparison to the natural vegetation surrounding the wetlands?

## Material and methods

**Study area**

**Figure 1.**
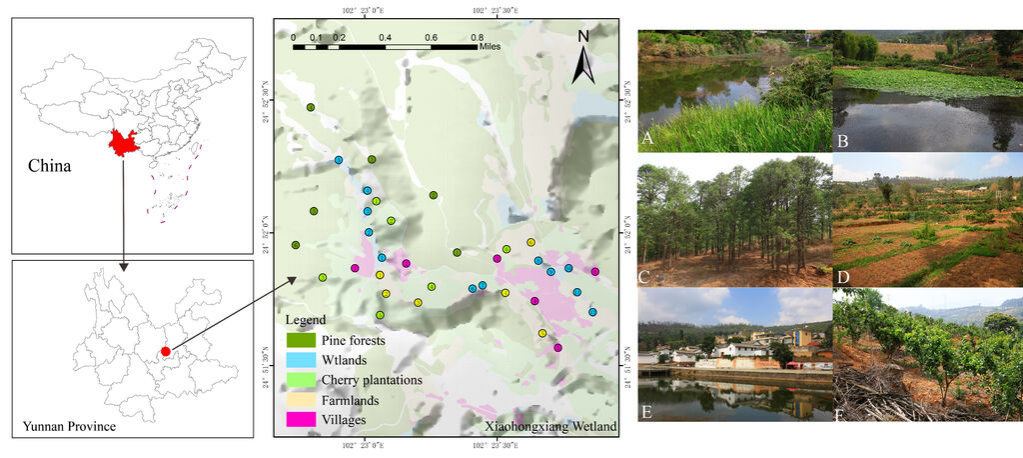
Habitats of Xiaohongxiang: **A**: Wetlands; **B**: Wetlands; **C**: Pine forests; **D**: Farmlands; **E**: Villages; **F**: Cherry plantations.

Fieldwork was conducted in Xiaohongxiang (102°21′-102°23′E, 24°51′-24°53′N) located in Anning City, Yunnan Province, China. The study area has an elevation of 1,900 to 2,000 m above sea level. Xiaohongxiang belongs to a typical subtropical climate in the mid-latitude high-altitude area, characterised by moderate seasonal temperature changes and a clear distinction between dry and wet seasons. The average annual temperature is 14.8℃ and precipitation is about 877.4 mm, with most rainfall concentrated from May to September. Xiaohongxiang mainly consists of ten reservoirs formed by dams, with a total area of 6.67 ha, the individual areas ranging from 0.06 to 2.12 ha. Xiaohongxiang includes five typical habitats: wetlands, villages, farmlands, cherry plantations and pine forests (Fig. 1). The predominant plant species found in the wetlands include *Cyperusinvolucratus*, *Nelumbonucifera*, *Phragmitesaustralis*, *Persicariahydropiper* and *Typhaorientalis*. The villages and farmlands constitute a human-modified habitat, the dominant plants in the villages including *Photiniaglomerata*, *Koelreuteriapaniculata*, *Toonasinensis* and *Diospyroskaki*. Farmlands predominantly cultivate corn and soybeans, with a small number of cherry and eucalyptus trees also interspersed. Cherry plantations are categorised as artificial plantations and primarily serve the purposes of promoting tourism development, as well as contributing to water and soil conservation efforts. The pine forests are a component of the natural vegetation, predominantly composed of *Keteleeriaevelyniana* and *Pinusyunnanensis*, the area also containing a small number of *Alnuscremastogyne*, *Micheliayunnanensis* and *Berberisjamesiana*.

**Sampling plots**

We conducted a bird survey using the point count method (Bibby et al. 1992). Wetland habitats had twelve sampling points, while farmlands, cherry plantations, pine forests and villages each had six sampling points, totalling thirty-six points. The monitoring period was extended from November 2023 to June 2024, encompassing both the breeding and non-breeding seasons of bird species. In the sampling design, the spacing between sampling points varied from 100 m to 200 m and each point was observed for a duration of 10 to 15 minutes. The sampling points were surveyed every month, resulting in a total of eight surveys being conducted. The survey times were scheduled for the morning (7:00-10:00 h) and afternoon (17:00-19:00 h), during which bird species observed or heard within a 50 m radius of the sampling points are meticulously documented using binoculars. Surveys were avoided during adverse weather conditions such as rain or heavy fog. Bird species identification follows Zheng (2023), while their threatened status is referenced against the IUCN Red List of Threatened Species (Http://www.iucnredlist.org).

**Data analysis**

Initially, we conducted an assessment of the adequacy of bird sampling by utilising the "iNEXT" package in R to generate rarefaction/extrapolation curves, based on sample size and coverage (Chao et al. 2014, Hsieh et al. 2016). A sample coverage exceeding 0.90 indicates sufficient sampling (Song et al. 2020). We assessed bird diversity through species richness, the Shannon-Wiener diversity index and the Simpson index following Hill numbers (Chao et al. 2014), where "q = 0" represents species richness, "q = 1" represents the Shannon-Wiener index reflecting common species richness and "q = 2" represents the Simpson index indicating dominant species richness (Chao et al. 2014). We used the Bray-Curtis distance metric to assess the similarity in bird species composition across diverse habitats. In this study, as accurately identifying the specific habitat types utilised by terrestrial bird species active in the riparian zones poses a significant challenge, we have excluded these birds from our analysis of bird diversity in different habitats. These species include White Wagtail (*Motacillaalba*), Grey Wagtail (*Motacillacinerea*), Black-capped Kingfisher (*Halcyonpileata*) and Black Kite (*Milvusmigrans*).

Secondly, for each documented bird species, we acquired data on five functional traits from Wang et al. (2021) that characterise the niche of the species, providing insights into how species utilise and compete for resources within their respective habitats (Luck et al. 2012). We used the following traits: body mass, bill length, tarsus length, wing length and feeding guild (insectivores, carnivores, nectarivorous, granivores and omnivores). Amongst these traits, body mass, bill length, tarsus length and wing length are continuous variables. Feeding guild is a categorical variable.

We calculated the indices of functional richness (FRic), functional divergence (FDiv), functional dispersion (FDis) and functional evenness (FEve), respectively. Functional diversity is defined as follows (Mason et al. 2005, Villéger et al. 2008, Mouchet et al. 2010, Laliberté and Legendre 2010): functional richness measures the extent of functional space utilised by a group of species. Functional evenness indicates the uniformity of species abundances across functional space. Functional divergence quantifies the distance between high species abundances and the centre of functional space. The three indices complement each other. Additionally, functional divergence, functional evenness and functional dispersion are not influenced by species richness, allowing for unbiased comparisons of communities with varying species richness (Villéger et al. 2008). Lastly, functional dispersion measures the average distance of each species to the centre of all species, taking into account their respective weights. The analysis of functional diversity was conducted using the Gower distance in the R "FD" package (Gower 1971, Villéger et al. 2008, Laliberté and Legendre 2010).

Thirdly, the scientific names of species were provided by us to facilitate the generation of a phylogeny from megatrees (see details in Li (2023)). Based on the bird species checklist obtained from our survey, we can construct a phylogenetic tree by grouping species into families/genera using supertrees. We constructed a phylogenetic tree using the BirdTree database (http://birdtree.org) (Jetz et al. 2012, Li 2023) (Suppl. material 5). This phylogenetic tree enables the calculation of Faith’s index of phylogenetic diversity (PD, Faith (1992)), mean pairwise distance (MPD) and mean nearest taxon distance (MNTD) for analysis of phylogenetic diversity (Webb 2000). To quantify phylogenetic patterns in community structure, a null model using an independent swapping algorithm can be used to stochastically generate species richness and occurrence frequencies (Manly 1995). Subsequently, the mean FD, MPD and MNTD values for the null model were calculated and then compared with the observed values (Zhao et al. 2020). The standard effect size (SES) was calculated using the following formula:

SES=（*M_obs_* -*M_null_*）/*SD_null_*

*M_obs_* is the observed value of PD/MPD/MNTD. *M_null_* is the average of the 999 null model PD/MPD/MNTD values generated randomly. *SD_null_* is the standard deviation of the 999 random values. A negative SES.MPD/MNTD indicates a clustered community phylogenetic structure, whereas a positive SES suggests an overdispersion structure (Webb 2000). SES.MPD or SES.MNTD greater than 1.96 (*p* < 0.05) signifies a significant overdispersion community structure, potentially attributed to competitive exclusion. Conversely, an SES less than -1.96 (*p* < 0.05) indicates a significantly clustered community structure, likely influenced by environmental filtering. An SES falling within the range of -1.96 to 1.96 (*p* > 0.05) implies that the community assembly follows a random process (Webb 2000, Zhao et al. 2020). The aforementioned analysis was conducted using the R package "picante" and "rtrees".

Finally, we used the non-parametric Kruskal-Wallis rank sum test to compare the taxonomic, functional and phylogenetic diversities of birds across different habitats. Then, we conducted post-hoc pairwise comparisons using Dunn's test to identify specific habitats that exhibit significant differences from each other, with p-values being adjusted with the Bonferroni method. Data analysis was performed in R (R Core Team 2024).

## Results

**Species composition**

**Figure 2.**
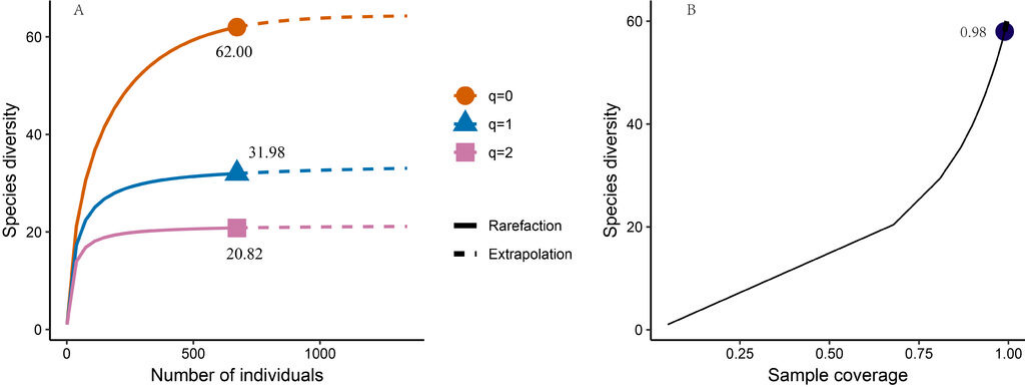
Bird species diversity in Xiaohongxaing. **A** "q = 0" represents species richness, "q = 1" represents the Shannon-Wiener index reflecting common species, "q = 2" represents the Simpson index indicating dominant species; **B** Sample coverage.

The assessment of species sampling adequacy shows a sample coverage of 0.98 (Fig. 2B). We recorded 62 bird species belonging to 35 families and 12 orders (Fig. 2A) (Suppl. material 1). None of the species was identified as IUCN-threatened bird species. We recorded a total of seven waterbird species, namely: Black-tailed Crake (*Amaurornisbicolor*), Little Egret (*Egrettagarzetta*), Little Grebe (*Tachybaptusruficollis*), Grey Heron (*Ardeacinerea*), White-breasted Waterhen (*Amaurornisphoenicurus*), Kentish Plover (*Charadriusalexandrinus*) and Common Moorhen (*Gallinulachloropus*). Four bird species without clearly defined habitats were omitted, resulting in a total of 58 observed bird species, the dominant species including Brown-breasted Bulbul (*Hirundorustica*), Sooty-headed Bulbul (*Pycnonotusaurigaster*), Eurasian Tree Sparrow (*Passermontanus*), Great Tit (*Parusmajor*), Scaly-breasted Munia (*Lonchurapunctulata*) and White-browed Laughingthrush (*Garrulaxsannio*).

**Figure 3.**
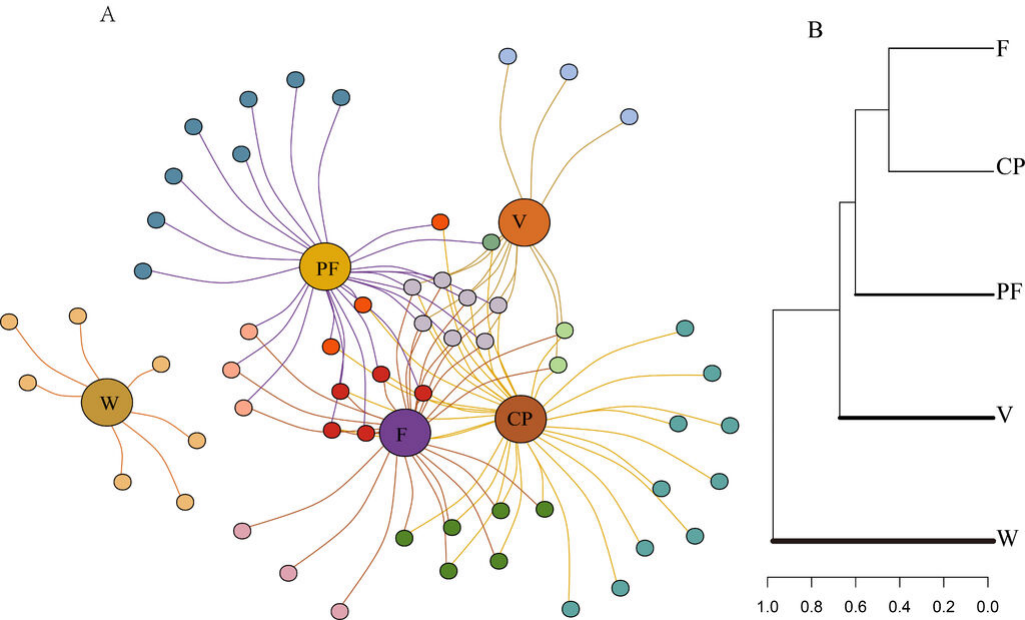
The composition of bird species is similar across different habitats. **A** Species habitat networks; **B** The similarity of bird species composition. **Habitats**: V (Villages), F (Farmlands), W (Wetlands), CP (Cherry plantations), PF (Pine forests). Different circles represent different species, different colours representing different habitats.

**Figure 4.**
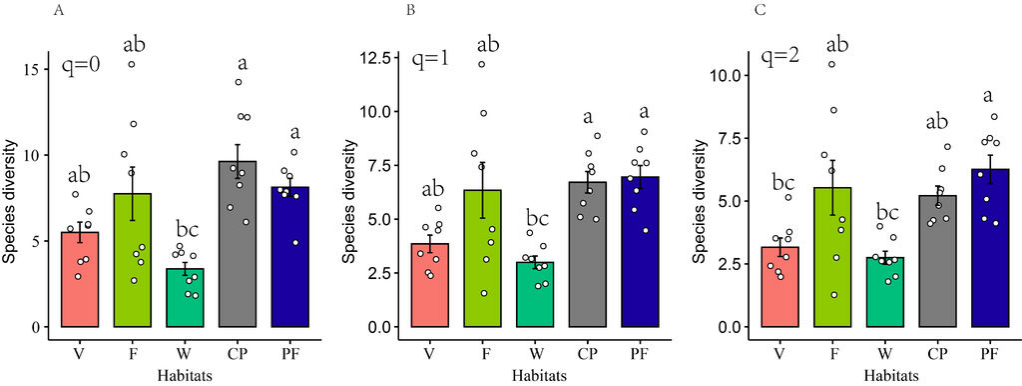
Comparison of species taxonomic diversity amongst different habitats. **A** "q = 0" represents species richness; **B** "q = 1" represents the Shannon-Wiener index reflecting common species; **C** "q = 2" represents the Simpson index indicating dominant species. **Habitats**: V (Villages), F (Farmlands), W (Wetlands), CP (Cherry plantations), PF (Pine forests). Different letters indicate significant differences and the same letters indicate no significant differences.

Unsurprisingly, no single habitat encompasses all the bird species (Fig. 3A) and the composition of bird species in the wetland habitat differs significantly from that in the other habitats. Conversely, there is a greater similarity in bird species composition amongst the farmlands, villages, cherry plantations, and pine forests (Fig. 3B). The analysis of bird species richness across various habitats reveals that cherry plantations exhibit the highest bird species richness, followed by pine forests, farmlands, villages and wetlands (Fig. 4A) (Suppl. material 2). The disparity in bird species richness between wetlands and cherry plantations (*z* = -3.95, *p* < 0.001)/pine forests (*z* = -3.37, *p* = 0.007) is statistically significant. The Shannon-Wiener index of pine forests is the highest and the lowest is in wetlands, with the difference between habitats consistent with the results of species richness (Fig. 4B). The Simpson index of pine forests is the highest and the lowest is in wetlands (Fig. 4C). Pine forests were significantly different from wetlands (*z* = -3.63, *p* = 0.002) and villages (*z* = 3.21, *p* = 0.01).

**Functional diversity**

**Figure 5.**
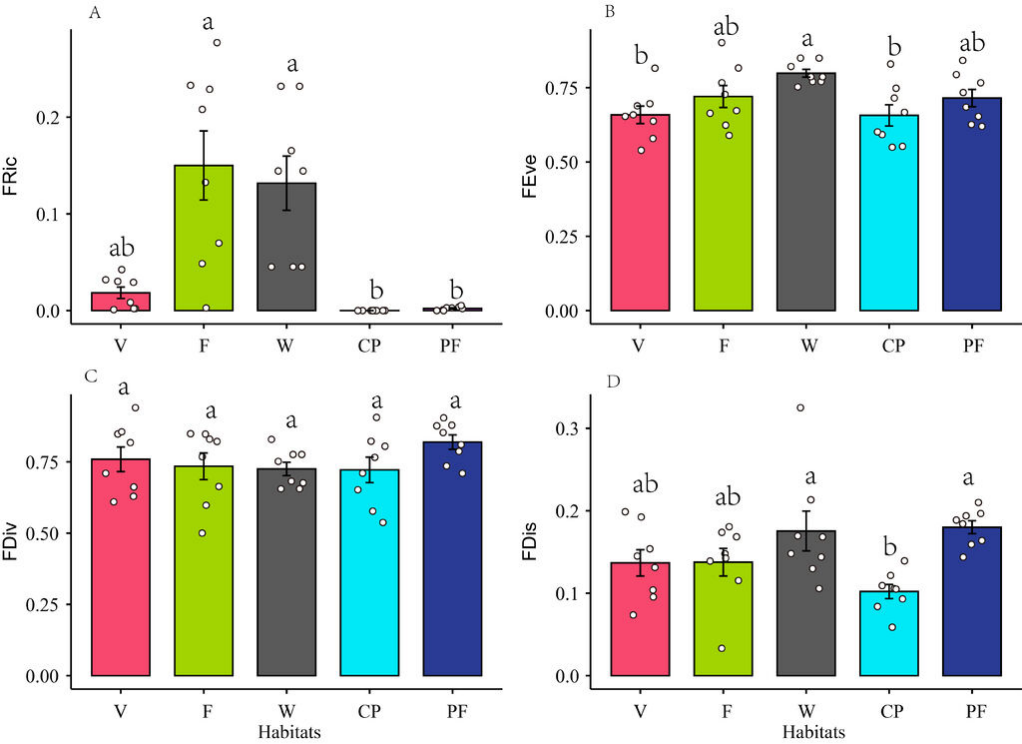
Comparison of species functional diversity amongst different habitats. **A** Functional richness (FRic); **B** Functional evenness (FEve); **C** Functional divergence (FDiv); **D** Functional dispersion (FDis). **Habitats**: V (Villages), F (Farmlands), W (Wetlands), CP (Cherry plantations), PF (Pine forests). Different letters indicate significant differences and the same letters indicate no significant differences.

The analysis of functional diversity revealed that functional richness was highest in farmland and wetlands, while it was lowest in cherry plantations and pine forests (Fig. 5A) (Suppl. material 3). There was a significant difference in functional richness between farmlands and cherry plantations (*z* = 4.59, *p <* 0.001) and wetlands and cherry plantations (*z* = 4.57, *p <* 0.001). There was no significant difference between cherry plantations and pine forests (*z* = 1.54, *p* = 1.00) (Fig. 5A). Functional evenness was highest in wetlands, with a significant difference from the villages (*z* = -2.99, *p* = 0.03) and cherry plantations (*z* = 2.99, *p* = 0.03) (Fig. 5B). The functional divergence between habitats shows no discernible difference (Fig. 5C). Furthermore, there was a significant disparity in functional dispersion between cherry plantations and pine forests (*z* = 3.74, *p* = 0.002) (Fig. 5D).

**Phylogenetic diversity**

**Figure 6.**
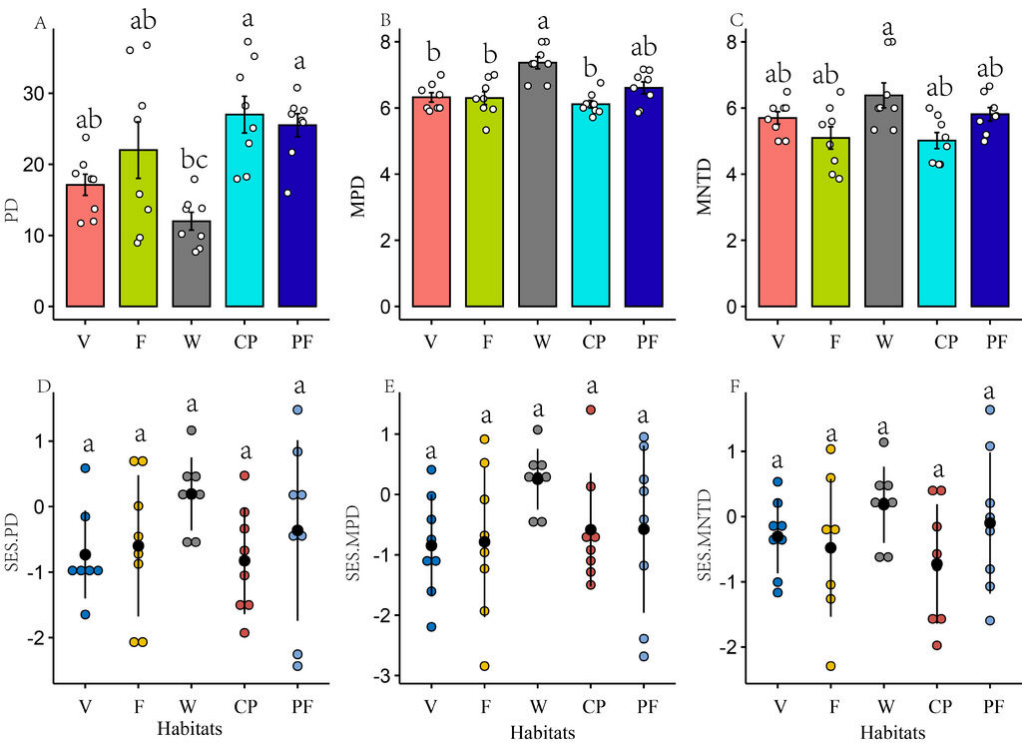
Comparison of species phylogenetic diversity amongst different habitats. **A** Phylogenetic diversity (PD); **B**: Mean pairwise distance (MPD); **C** Mean nearest taxon distance (MNTD); **D** Standard effect size of PD (SES.PD); **E** Standard effect size of MPD (SES.MPD); **F** Standard effect size of MNTD (SES.MNTD). **Habitats**: V (Villages), F (Farmlands), W (Wetlands), CP (Cherry plantations), PF (Pine forests). Different letters indicate significant differences and the same letters indicate no significant differences.

Phylogenetic diversity showed that the highest PD was found in cherry plantations (*z* = -5.18, *p <* 0.001) and pine forests (*z* = -4.32, *p <* 0.001), which was significantly different from wetlands (Fig. 6A) (Suppl. material 4). In addition, cherry plantations were significantly different from farmlands (*z* = -3.13, *p* = 0.02) and villages (*z* = -3.13, *p* = 0.02). SES.PD did not differ significantly amongst different habitats (Fig. 6D). However, the highest MPD was found in cherry plantations (*z* = -5.36, *p <* 0.001) and pine forests (*z* = -3.88, *p* = 0.001), which were significantly different from wetlands (Fig. 6B). SES.MPD did not differ significantly amongst different habitats (Fig. 6E). Similarly, the highest MNTD was found in pine forests, there being a significant difference from wetlands (*z* = -3.02, *p* = 0.03) (Fig. 6C). SES.MNTD did not differ significantly amongst different habitats (Fig. 6F).

## Discussion

**Waterbirds species of small wetlands**

In general, small wetlands play a crucial role by providing vital habitats for waterbirds, serving as key locations for breeding, nesting and resting (Wang et al. 2023, Li et al. 2024). We only have seven waterbird species, these species being prevalent inhabitants of China's extensive wetlands. Wang et al. (2021) demonstrated that anthropogenic landscapes (such as ponds, impoundments and reservoirs) have a positive effect on the population maintenance of common waterbird species. Nevertheless, for rare waterbird species, anthropogenic landscapes did not demonstrate the same positive effects. In addition, artificial wetlands have a consistently lower value than restored and natural wetlands (Sebastián‐González and Green 2016, Almeida et al. 2020). While natural wetlands are essential for threatened species, restored wetlands can be of similar value and can ensure the maintenance of key ecological processes (Sebastián‐González and Green 2016). Our findings provide empirical evidence of the limited ability of small wetlands to protect threatened or rare waterbirds. For all that, the composition of wetland birds was different from that of birds in farmlands, villages, cherry plantations and pine forests. Consequently, small wetlands still hold great value in the conservation of common species.

**Cherry plantations and pine forests had higher bird species richness**

Bird species richness and abundance are found to be negatively impacted by plantations, although there is considerable variation amongst different study cases (Calviño-Cancela 2013, Castaño-Villa et al. 2019). Curiously, bird richness was highest in cherry plantations, while the Shannon Wiener and Simpson indices were higher in pine forests. For bird species richness, our findings were inconsistent with previous studies in southern Europe (Reino et al. 2009) and southern America (Zurita et al. 2006). In Morocco, natural forests exhibited higher bird diversity compared to both olive and eucalyptus plantations (Hanane et al. 2018). Similarly, bird species richness was highest in native forests and lowest in shrublands and eucalyptus plantations in Spain (Calviño-Cancela 2013). We recorded several dominant bird species in cherry plantations and pine forests, including the Brown-breasted Bulbul, Oriental White-eye (*Zosteropspalpebrosus*), White-browed Laughingthrush, Crested Finchbill (*Spizixoscanifrons*), Great Tit etc. From a practical perspective, the higher richness in plantations and pine forests can be attributed to the presence of understorey vegetation, cherry fruit and insects, which provide foraging and breeding grounds for these species. Additionally, cherry plantations may have better connectivity with the surrounding natural ecosystems than farmlands and villages, which facilitates the migration and dispersal of birds between different habitats. Kavanagh et al. (2007) demonstrated that replanting with eucalyptus trees resulted in significant increases in bird population density compared to paddocks that had been cleared or had few trees. Furthermore, areas with a mix of eucalyptus and shrub plantings supported bird communities similar to those found in the remaining native forests and woodland in the area. This suggests that maintaining connectivity between forest plantations and native remnants may also enhance the richness of bird species (Calviño-Cancela 2013).

**Contrasting patterns of species richness and functional diversity**

Functional richness typically remains stable or increases as species richness increases (Villéger et al. 2008, Mouchet et al. 2010). When comparing cherry plantations and pine forests to wetlands and farmlands, the functional richness of the former is notably lower. This low functional richness suggests that some resources within the community are underutilised, which may lead to decreased resilience to environmental fluctuations (Mason et al. 2005). Cherry plantations and pine forests have inherent limitations in sustaining the functional diversity of bird communities. The plantations, however, have significantly diminished the functional diversity of birds, aligning our findings with previous studies (Corbelli et al. 2015, Jacoboski et al. 2016). However, our findings contrast with those from south-eastern South America (Jacoboski and Hartz 2020) and the Atlantic Forest of South America (Vaccaro et al. 2022). In practice, this discrepancy may be attributed to the simplified vegetation structure and reduced diversity in monoculture plantations and single-species natural forests, which could limit the birds' ability to exploit a variety of ecological niches (Hua et al. 2022). In addition, in plantations, habitat generalists replace specialists, leading to functional homogenisation (Almeida et al. 2018, Jacoboski and Hartz 2020).

In this study, although the wetland bird species were relatively few, they had a high functional richness. This suggests that these waterbirds have different ecological roles and functions (Ma et al. 2009). It also suggests that the degree of ecological space utilisation is higher in wetlands (Mason et al. 2005, Villéger et al. 2008). Previous studies have demonstrated that the presence of wetlands can significantly enhance the functional diversity of bird communities (Zhao et al. 2023). Farmlands have the highest bird functional richness, which may be related to the fact that the study area has a high level of landscape diversity in farmland. Heterogeneous farmland habitats can provide a greater niche space, thereby increasing the species diversity and functional richness of birds (Morelli 2018). Functional richness and evenness are independent of each other (Mason et al. 2005, Villéger et al. 2008). Generally, in environments where the habitat is disturbed and the vegetation structure is simple, birds tend to exhibit higher functional evenness (Schleuter et al. 2010, Marcacci et al. 2021). However, there are also studies indicating a decrease in functional evenness levels following disturbance (Mouillot et al. 2013). Lower functional evenness suggests that certain areas of the niche space are not fully utilised, which can lead to a decrease in overall productivity (Mason et al. 2005). Functional evenness decreases when species abundance is unevenly distributed or when there is an irregularity in functional distances amongst species, similar to how species evenness pertains solely to the abundances of present species (Mason et al. 2005, Villéger et al. 2008). Hence, the functional evenness contribution of each species is directly related to its abundance (Villéger et al. 2008). Compared to villages and farmlands, there is a higher functional evenness of birds in the wetlands. In this context, no single waterbird species became the dominant species in Xiaohongxiang, with individual species abundance ranging from one to three individuals. Consequently, the low species richness and relatively even abundance of wetland birds may have led to higher functional evenness.

Functional divergence was not associated with functional richness or evenness (Mason et al. 2005). A high level of functional divergence suggests a significant degree of niche differentiation, promoting reduced competition for resources. Therefore, communities exhibiting high functional divergence may potentially enhance ecosystem function through the more efficient utilisation of resources (Mason et al. 2005). The same level of functional divergence suggests that the birds in different habitats maintain similar diversity in resource utilisation patterns since the distribution of species in function trait space is similar (Mason et al. 2005, Jacoboski and Hartz 2020). Functional dispersion quantifies the distribution of traits based on their range values (Laliberté and Legendre 2010). Species with similar traits will accumulate at sites where strong filtering occurs, resulting in low functional dispersion, whereas higher dispersion indicates an increased trait diversity and niche partitioning (Dietzel et al. 2024). In general, an environmental disturbance may result in a reduction of functional dispersion (Mouillot et al. 2013). There is a significant disparity in bird functional dispersion between wetlands and pine forests, as well as cherry plantations and pine forests. Cherry plantations with lower functional dispersion may be associated with a few generalised species occupying more trait space (Bleuel et al. 2024). In addition, it may also be related to landscape diversity. Dietzel et al. (2024) demonstrated a positive association between functional dispersion and landscape diversity, suggesting the presence of trait-based habitat filtering. Raposo et al. (2023) demonstrated that landscape changes over time adversely affect functional dispersion. Our results suggest that converting wetlands and pine forest landscapes into cherry plantations may result in a reduction in functional dispersion and ecosystem functioning (Barbaro et al. 2013, Prescott et al. 2016).

**Cherry plantations had higher phylogenetic diversity**

The highest PD was observed in cherry plantations and pine forests, a finding that significantly differed from the PD observed in wetlands. As is widely acknowledged, there was a significant correlation between PD and species richness (Miller et al. 2016, Tucker et al. 2016). It was observed that wetland birds exhibited the lowest species richness, resulting in lower PD, which is consistent with the findings of the comparison of species richness across different habitats. The impact of species richness can be mitigated through the application of null models (Pavoine et al. 2013). However, SES.PD, SES.MPD and SES.MNTD did not differ significantly amongst different habitats, the finding indicating that the PD of the wetland is higher than expected. The variations of PD, MPD and MNTD may indicate that taxa are associated with different habitats at various phylogenetic levels (Webb 2000). Generally, the variation in the phylogenetic diversity of birds is influenced by a variety of factors, including plant species richness, canopy height, tree density and topographical elevation (Rurangwa et al. 2022). However, Bae et al. (2018) showed that the phylogenetic diversity of bird communities is inversely associated with productivity and heterogeneity in temperate forests. Our findings highlight the need for a more nuanced understanding of how habitat characteristics interact with phylogenetic diversity.

The detection of habitat filtering was most effective with PD and MNTD, while MPD metrics were most effective at identifying competitive exclusion (Miller et al. 2016, Miller et al. 2018). Receiving strong competitive exclusion, local communities may exhibit widespread phylogenetic overdispersion, with certain species being generally excluded from plots and possibly absent from the entire community. In contrast, habitat filtering is the process by which only species with similar traits can survive and reproduce within a given abiotic environment (Violle et al. 2011，Miller et al. 2016, Lv et al. 2024). In this study, our findings indicate that there was no notable clustering or overdispersion observed in the phylogenetic structure of bird species across all habitats. In other words, competitive exclusion, habitat filtering and neutral processes may collectively influence bird community assembly in the study area. Our results are consistent with those of Jia et al. (2020) and Yuan et al. (2021). To some extent, the processes underlying the emergence of community phylogenetic patterns vary across different spatial scales (Vamosi et al. 2009, Gerhold et al. 2015). On the other hand, the presence of species in smaller geographical areas can be reflected in the phylogeny of the overall species pool. The arrangement of these species on the phylogeny may show clustering, randomness or dispersion across the entire pool (Webb 2000). Therefore, the indication of non-random spatial correlation between species and their habitats is an important, but not completely adequate factor in evaluating the importance of habitat partitioning in promoting co-existence amongst multiple species.

## Conclusions

The study specifically investigated the impact of Xiaohongxiang and its surrounding environments on bird diversity. Despite their importance, small wetlands tend to exhibit lower levels of species richness and phylogenetic diversity. Our findings demonstrate that these habitats, crucial for sustaining common waterbird species, may not provide adequate protection for threatened or rare species. Although cherry plantations and pine forests contribute positively to avian diversity in the vicinity of wetlands, the observed low functional diversity implies that monoculture plantations could be limiting the full range of ecological niches available to birds. This suggests that the structural simplicity of monocultures may restrict the variety of habitats and resources necessary for a diverse bird community. Although our study was constrained by a limited sample size, which may affect the robustness of these observations, the results underscore a notable discrepancy between taxonomic and functional diversity amongst bird species. This discrepancy indicates that reliance on taxonomic diversity alone may not fully capture the ecological complexity of bird communities. Therefore, employing multidimensional biodiversity metrics can offer a more nuanced and comprehensive assessment of the impacts of wetland restoration projects and the surrounding habitats on avian diversity.

## Supplementary Material

2208282E-07DE-580A-B41D-0600C5BC5DAE 10.3897/BDJ.12.e136248.suppl1 Supplementary material 1 List of bird species in Xiaohongxiang Data type Table File: oo_1121306.xls https://binary.pensoft.net/file/1121306 Binqiang Li

62D6885C-B201-58D6-A095-B9642723C846 10.3897/BDJ.12.e136248.suppl2 Supplementary material 2 Comparison of species taxonomic diversity amongst different habitats Data type Table File: oo_1121058.xls https://binary.pensoft.net/file/1121058 Binqiang Li

DFF41F46-EB1D-57BB-A96D-660581DBD069 10.3897/BDJ.12.e136248.suppl3 Supplementary material 3 Comparison of species functional diversity amongst different habitats Data type Table File: oo_1121061.xls https://binary.pensoft.net/file/1121061 Binqiang Li

0EE932A6-9717-5360-96CF-7A6984400871 10.3897/BDJ.12.e136248.suppl4 Supplementary material 4 Comparison of species phylogenetic diversity amongst different habitats Data type Table File: oo_1121065.xls https://binary.pensoft.net/file/1121065 Binqiang Li

C3AE24AF-3D23-53D0-A63A-1AB9ACA5DFFA 10.3897/BDJ.12.e136248.suppl5 Supplementary material 5 Phylogenetic tree of the 58 bird species in the Xiaohongxiang Wetland Data type images File: oo_1177035.jpg https://binary.pensoft.net/file/1177035 Binqiang Li

## References

[B12005132] Almeida Bia A., Sebastián‐González Esther, dos Anjos Luiz, Green Andy J. (2020). Comparing the diversity and composition of waterbird functional traits between natural, restored, and artificial wetlands. Freshwater Biology.

[B12005142] Almeida Sara Miranda, Juen Leandro, Sobral Fernando Landa, Santos Marcos Pérsio Dantas (2018). The influence of biogeographic history on the functional and phylogenetic diversity of passerine birds in savannas and forests of the Brazilian Amazon. Ecology and Evolution.

[B12005151] Bae Soyeon, Müller Jörg, Lee Dowon, Vierling Kerri T., Vogeler Jody C., Vierling Lee A., Hudak Andrew T., Latifi Hooman, Thorn Simon (2018). Taxonomic, functional, and phylogenetic diversity of bird assemblages are oppositely associated to productivity and heterogeneity in temperate forests. Remote Sensing of Environment.

[B12005165] Barbaro Luc, Giffard Brice, Charbonnier Yohan, van Halder Inge, Brockerhoff Eckehard G. (2013). Bird functional diversity enhances insectivory at forest edges: a transcontinental experiment. Diversity and Distributions.

[B12005175] Bega João M. M., Saltarelli Wesley A., Gücker Björn, Boëchat Iola G., Finkler Nicolas R., Cunha Davi G. F. (2024). Effects of riparian vegetation restoration and environmental context on ecosystem functioning in tropical streams of southeastern Brazil. Science of The Total Environment.

[B12005186] Bibby Colin J., Burgess Neil D., Hill David A. (1992). Point Counts. Bird Census Techniques.

[B12005195] Bleuel Jessica, Waechter Luiza, Bender Mariana, Longo Guilherme O. (2024). Taxonomic and functional diversity of zooxanthellate corals and hydrocorals in Southwestern Atlantic reefs. Frontiers in Ecology and Evolution.

[B12005204] Calviño-Cancela María (2013). Effectiveness of eucalypt plantations as a surrogate habitat for birds. Forest Ecology and Management.

[B12005213] Castaño-Villa Gabriel J., Estevez Jaime V., Guevara Giovany, Bohada-Murillo Mauricio, Fontúrbel Francisco E. (2019). Differential effects of forestry plantations on bird diversity: A global assessment. Forest Ecology and Management.

[B12005223] Chao Anne, Gotelli Nicholas J., Hsieh T. C., Sander Elizabeth L., Ma K. H., Colwell Robert K., Ellison Aaron M. (2014). Rarefaction and extrapolation with Hill numbers: a framework for sampling and estimation in species diversity studies. Ecological Monographs.

[B12005235] Corbelli Julian Martin, Zurita Gustavo Andres, Filloy Julieta, Galvis Juan Pablo, Vespa Natalia Isabel, Bellocq Isabel (2015). Integrating taxonomic, functional and phylogenetic beta diversities: Interactive effects with the biome and land use across taxa. PLOS ONE.

[B12005246] Cui L. J, Lei Y. R, Zhang M. Y, Li W (2021). Review on small wetlands: definition, typology and ecological services. Acta Ecologica Sinica.

[B12005258] Davidson N. C., van Dam A. A., Finlayson C. M., McInnes R. J. (2019). Worth of wetlands: revised global monetary values of coastal and inland wetland ecosystem services. Marine and Freshwater Research.

[B12005267] Devictor Vincent, Mouillot David, Meynard Christine, Jiguet Frédéric, Thuiller Wilfried, Mouquet Nicolas (2010). Spatial mismatch and congruence between taxonomic, phylogenetic and functional diversity: the need for integrative conservation strategies in a changing world. Ecology Letters.

[B12005278] Dietzel Simon, Rojas-Botero Sandra, Dichtl Anja, Kollmann Johannes, Fischer Christina (2024). Winners and losers at enhanced urban roadsides: Trait-based structuring of wild bee communities at local and landscape scale. Biological Conservation.

[B12005288] Faith Daniel P. (1992). Conservation evaluation and phylogenetic diversity. Biological Conservation.

[B12005297] Fluet-Chouinard Etienne, Stocker Benjamin D., Zhang Zhen, Malhotra Avni, Melton Joe R., Poulter Benjamin, Kaplan Jed O., Goldewijk Kees Klein, Siebert Stefan, Minayeva Tatiana, Hugelius Gustaf, Joosten Hans, Barthelmes Alexandra, Prigent Catherine, Aires Filipe, Hoyt Alison M., Davidson Nick, Finlayson C. Max, Lehner Bernhard, Jackson Robert B., McIntyre Peter B. (2023). Extensive global wetland loss over the past three centuries. Nature.

[B12005323] Gerhold Pille, Cahill James F., Winter Marten, Bartish Igor V., Prinzing Andreas (2015). Phylogenetic patterns are not proxies of community assembly mechanisms (they are far better). Functional Ecology.

[B12005333] Gower J. C. (1971). A general coefficient of similarity and some of its properties. Biometrics.

[B12005342] Hanane S., Cherkaoui S. I., Magri N., Yassin M. (2018). Bird species richness in artificial plantations and natural forests in a North African agroforestry system: assessment and implications. Agroforestry Systems.

[B12005351] Hsieh T. C., Ma K. H., Chao Anne (2016). iNEXT: an R package for interpolation and extrapolation of species diversity (Hill numbers). Methods in Ecology and Evolution.

[B12005360] Htay Thazin, Røskaft Eivin, Ringsby Thor Harald, Ranke Peter Sjolte (2023). Spatio-temporal variation in avian taxonomic, functional, and phylogenetic diversity and its relevance for conservation in a wetland ecosystem in Myanmar. Biodiversity and Conservation.

[B12005369] Hua Fang Yuan, Bruijnzeel L. Adrian, Meli Paula, Martin Philip A., Zhang Jun, Nakagawa Shinichi, Miao Xinran, Wang Weiyi, McEvoy Christopher, Peña-Arancibia Jorge Luis, Brancalion Pedro H. S., Smith Pete, Edwards David P., Balmford Andrew (2022). The biodiversity and ecosystem service contributions and trade-offs of forest restoration approaches. Science.

[B12005406] Jacoboski L. I., Debastiani V. J., de Mendonça-Lima A., Hartz S. M. (2016). How do diversity and functional nestedness of bird communities respond to changes in the landscape caused by eucalyptus plantations?. Community Ecology.

[B12005415] Jacoboski Lucilene Inês, Hartz Sandra Maria (2020). Using functional diversity and taxonomic diversity to assess effects of afforestation of grassland on bird communities. Perspectives in Ecology and Conservation.

[B12005424] Jetz W., Thomas G. H., Joy J. B., Hartmann K., Mooers A. O. (2012). The global diversity of birds in space and time. Nature.

[B12005434] Jia Y. F, Zeng Qing, Wang Y. Y, Saintilan Neil, Lei G. C, Wen Li (2020). Processes shaping wintering waterbird communities in an intensive modified landscape: Neutral assembly with dispersal limitation and localized competition. Ecological Indicators.

[B12005447] Johnston Carol A. (2013). Wetland losses due to row crop expansion in the Dakota Prairie Pothole region. Wetlands.

[B12012017] Kavanagh Rodney p., Stanton Matthew a., Herring Matthew w. (2007). Eucalypt plantings on farms benefit woodland birds in south‐eastern Australia. Austral Ecology.

[B12005456] Laliberté Etienne, Legendre Pierre (2010). A distance‐based framework for measuring functional diversity from multiple traits. Ecology.

[B12005465] Li Dong, Xu He, Fan Chao, Wu Yang, Zhang Y,X, Hou X. Y (2024). Artificial wetlands providing space gain for the suitable habitat of coastal Pied Avocet. Estuarine, Coastal and Shelf Science.

[B12005494] Li D. J (2023). rtrees: an R package to assemble phylogenetic trees from megatrees. Ecography.

[B12005503] Liu L. R, Lin B. D, Fang Q. H, Jiang X. Y (2024). Effectiveness assessment of China's coastal wetland ecological restoration: A meta-analysis. Science of The Total Environment.

[B12005512] Luck Gary W., Lavorel Sandra, McIntyre Sue, Lumb Katrina (2012). Improving the application of vertebrate trait‐based frameworks to the study of ecosystem services. Journal of Animal Ecology.

[B12005521] Luo Kang, Wu Z. L, Bai H. T, Wang Z. J (2019). Bird diversity and waterbird habitat preferences in relation to wetland restoration at Dianchi Lake, south-west China. Avian Research.

[B12005530] Lv Ting, Ding Hui, Wang N. J, Xie Lei, Chen S. F, Wang Ding, Fang Y. M (2024). The roles of environmental filtering and competitive exclusion in the plant community assembly at Mt. Huangshan are forest-type-dependent. Global Ecology and Conservation.

[B12005551] Manly Bryan F. J. (1995). A note on the analysis of species co‐occurrences. Ecology.

[B12005560] Mao D. H, Luo Ling, Wang Z. M, Wilson Maxwell C., Zeng Yuan, Wu B. F, Wu J. G (2018). Conversions between natural wetlands and farmland in China: A multiscale geospatial analysis. Science of The Total Environment.

[B12012049] Marcacci Gabriel, Westphal Catrin, Wenzel Arne, Raj Varsha, Nölke Nils, Tscharntke Teja, Grass Ingo (2021). Taxonomic and functional homogenization of farmland birds along an urbanization gradient in a tropical megacity. Global Change Biology.

[B12005572] Mason Norman W. H., Mouillot David, Lee William G., Wilson J. Bastow (2005). Functional richness, functional evenness and functional divergence: The primary components of functional diversity. Oikos.

[B12005542] Ma Z. J, Cai Y. T, Li Bo, Chen J. K (2009). Managing wetland habitats for waterbirds: An international perspective. Wetlands.

[B12005581] Meli Paula, Rey Benayas José María, Balvanera Patricia, Martínez Ramos Miguel (2014). Restoration enhances wetland biodiversity and ecosystem service supply, but results are context-dependent: a meta-analysis. PLoS ONE.

[B12005599] Miller Eliot T., Farine Damien R., Trisos Christopher H. (2016). Phylogenetic community structure metrics and null models: a review with new methods and software. Ecography.

[B12005590] Miller Joseph T., Jolley‐Rogers Garry, Mishler Brent D., Thornhill Andrew H. (2018). Phylogenetic diversity is a better measure of biodiversity than taxon counting. Journal of Systematics and Evolution.

[B12005608] Morelli Federico (2018). High nature value farmland increases taxonomic diversity, functional richness and evolutionary uniqueness of bird communities. Ecological Indicators.

[B12005617] Mouchet Maud A., Villéger Sébastien, Mason Norman W. H., Mouillot David (2010). Functional diversity measures: an overview of their redundancy and their ability to discriminate community assembly rules. Functional Ecology.

[B12005626] Mouillot David, Graham Nicholas A. J., Villéger Sébastien, Mason Norman W. H., Bellwood David R. (2013). A functional approach reveals community responses to disturbances. Trends in Ecology & Evolution.

[B12005636] Mwita E., Menz G., Misana S., Becker M., Kisanga D., Boehme B. (2013). Mapping small wetlands of Kenya and Tanzania using remote sensing techniques. International Journal of Applied Earth Observation and Geoinformation.

[B12005647] Nakamura KEIGO, Tockner KLEMENT, Amano KUNIHIKO (2006). River and wetland restoration: Lessons from Japan. BioScience.

[B12005656] Owen Nisha R., Gumbs Rikki, Gray Claudia L., Faith Daniel P. (2019). Global conservation of phylogenetic diversity captures more than just functional diversity. Nature Communications.

[B12005665] Pavoine Sandrine, Gasc Amandine, Bonsall Michael B., Mason Norman W. H. (2013). Correlations between phylogenetic and functional diversity: mathematical artefacts or true ecological and evolutionary processes?. Journal of Vegetation Science.

[B12005691] Pei L. X., Ye S. Y., He L., Zhao G. M., Yuan H. M., Ding X. G., Pei S. F., Li X., Wang F. M., Laws E. A. (2023). Wetland resources, development and protection in China and management recommendations. Geology in China.

[B12005708] Prescott Graham . W., Gilroy James . J., Haugaasen Torbjørn, Medina U. C.A, Foster William . A., Edwards David. P. (2016). Managing Neotropical oil palm expansion to retain phylogenetic diversity. Journal of Applied Ecology.

[B12005719] Qiu Jie, Zhang Y. X, Ma J. W (2024). Wetland habitats supporting waterbird diversity: Conservation perspective on biodiversity-ecosystem functioning relationship. Journal of Environmental Management.

[B12005761] Raposo Karina Santos Paulinelli, Damasceno-Junior Geraldo Alves, Almeida-Gomes Mauricio, Araujo Andréa Cardoso (2023). The effects of urbanization on functional dispersion of plant reproductive traits in Cerrado fragments. Urban Ecosystems.

[B12005740] Team R Core (2024). R: a language and environment for statistical computing. r foundation for statistical computing, vienna, austria. https://www.R-project.org.

[B12005750] Reino Luís, Beja Pedro, Osborne Patrick E., Morgado Rui, Fabião António, Rotenberry John T. (2009). Distance to edges, edge contrast and landscape fragmentation: Interactions affecting farmland birds around forest plantations. Biological Conservation.

[B12006460] Rivera Gabriel, Gonzales Sergio, Aponte Héctor (2021). Wetlands of the South American pacific coast: a bibliometric analysis. Wetlands Ecology and Management.

[B12006471] Rurangwa Marie Laure, Niyigaba Protais, Tobias Joseph A., Whittaker Robert J. (2022). Functional and phylogenetic diversity of an agricultural matrix avifauna: The role of habitat heterogeneity in Afrotropical farmland. Ecology and Evolution.

[B12006481] Schleuter D., Daufresne M., Massol F., Argillier C. (2010). A user's guide to functional diversity indices. Ecological Monographs.

[B12012040] Sebastián‐González Esther, Green Andy J. (2016). Reduction of avian diversity in created versus natural and restored wetlands. Ecography.

[B12006490] Shen X. J, Jiang Ming, Lu X. G, Thompson Julian R. (2024). Protect and restore small wetlands. Science.

[B12006501] Sievers Michael, Hale Robin, Parris Kirsten M., Swearer Stephen E. (2017). Impacts of human‐induced environmental change in wetlands on aquatic animals. Biological Reviews.

[B12006510] Sinha Ankita, Hariharan Hima, Adhikari Bhupendra, Krishnamurthy Ramesh (2019). Bird diversity along riverine areas in the Bhagirathi Valley, Uttarakhand, India. Biodiversity Data Journal.

[B12006519] Song W. Y, Li X. Y, Chen Z. Z, Li Q, Onditi K. O, He S. W, Jiang X. L (2020). Isolated alpine habitats reveal disparate ecological drivers of taxonomic and functional beta-diversity of small mammal assemblages. Zoological Research.

[B12006537] Tucker Caroline M., Cadotte Marc W., Carvalho Silvia B., Davies T. Jonathan, Ferrier Simon, Fritz Susanne A., Grenyer Rich, Helmus Matthew R., Jin Lanna S., Mooers Arne O., Pavoine Sandrine, Purschke Oliver, Redding David W., Rosauer Dan F., Winter Marten, Mazel Florent (2016). A guide to phylogenetic metrics for conservation, community ecology and macroecology. Biological Reviews.

[B12006558] Vaccaro Anahí S., Filloy Julieta, Bellocq M. Isabel (2022). Bird taxonomic and functional diversity in urban settlements within a forest biome vary with the landscape matrix. Perspectives in Ecology and Conservation.

[B12006567] Vamosi S. M, Heard S. B, Vamosi J. C, Webb C. O (2009). Emerging patterns in the comparative analysis of phylogenetic community structure. Molecular Ecology.

[B12006576] Villéger Sébastien, Mason Norman W. H., Mouillot David (2008). New multidimensional functional diversity indices for a multifaceted framework in functional ecology. Ecology.

[B12006585] Violle Cyrille, Nemergut Diana R., Pu Zhichao, Jiang Lin (2011). Phylogenetic limiting similarity and competitive exclusion. Ecology Letters.

[B12006594] Wang Fang, Yang Y. B, Song Gang, Shi XJ, Pu Bu, Yang Le (2023). Mangcuo lake in hengduan mountains: an important alpine breeding and stopover site along central Asian flyway. Animals.

[B12006605] Wang X. D, Li X. H, Ren X. T, Jackson Micha V., Fuller Richard A., Melville David S., Amano Tatsuya, Ma Z. J (2021). Effects of anthropogenic landscapes on population maintenance of waterbirds. Conservation Biology.

[B12006618] Wang Y. P, Song Y. F, Zhong Y. X, Chen C. W, Zhao Y. H, Zeng Di, Wu Y. R, Ding Ping (2021). A dataset on the life-history and ecological traits of Chinese birds. Biodiversity Science.

[B12006632] Webb Campbell O. (2000). Exploring the phylogenetic structure of ecological communities: An example for rain forest trees. The American Naturalist.

[B12006641] Xiong Ying, Mo S. H, Wu H. P, Qu X. Y, Liu Y. Y, Zhou Lu (2023). Influence of human activities and climate change on wetland landscape pattern—A review. Science of The Total Environment.

[B12006652] Yuan S. J, Miao K. E, Sun W., Qian R. E, Wang H, Hu C. C., Chang Q (2021). Phylogenetic and functional structure of bird communities in Yancheng Jiulongkou wetland park. Journal of Nanjing Normal University.

[B12006664] Zedler Joy B., Kercher Suzanne (2005). Wetland resources: status, trends, ecosystem services, and restorability. Annual Review of Environment and Resources.

[B12006673] Zhang T. X, Chen Xue, Wu Y. J, Ran J. H (2020). Diversity and structure of bird communities in contrasting forests of the Hengduan Mountains, China. Biodiversity and Conservation.

[B12006682] Zhao Y. H, Dunn Robert R., Zhou H. N, Si X. F, Ding Ping (2020). Island area, not isolation, drives taxonomic, phylogenetic and functional diversity of ants on land‐bridge islands. Journal of Biogeography.

[B12006692] Zhao Z. X, Borzée Amaël, Li J. H, Chen Sheng, Shi Hui, Zhang Yong (2023). Urban bird community assembly mechanisms and driving factors in university campuses in Nanjing, China. Animals.

[B12006703] Zheng G. A (2023). A checklist on the classification and distribution of the birds of China. Science Press, Beijing.

[B12006714] Zurita G. A., Rey N., Varela D. M., Villagra M., Bellocq M. I. (2006). Conversion of the Atlantic Forest into native and exotic tree plantations: Effects on bird communities from the local and regional perspectives. Forest Ecology and Management.

