# Supplementary figures and images for: The taxonomic, functional and phylogenetic diversity of birds in Xiaohongxiang Wetland, southwest China
Source: Biodivers Data J. 2024 Dec 4;12:e136248. doi: 10.3897/BDJ.12.e136248 (PMC11635359; doi:10.3897/BDJ.12.e136248)

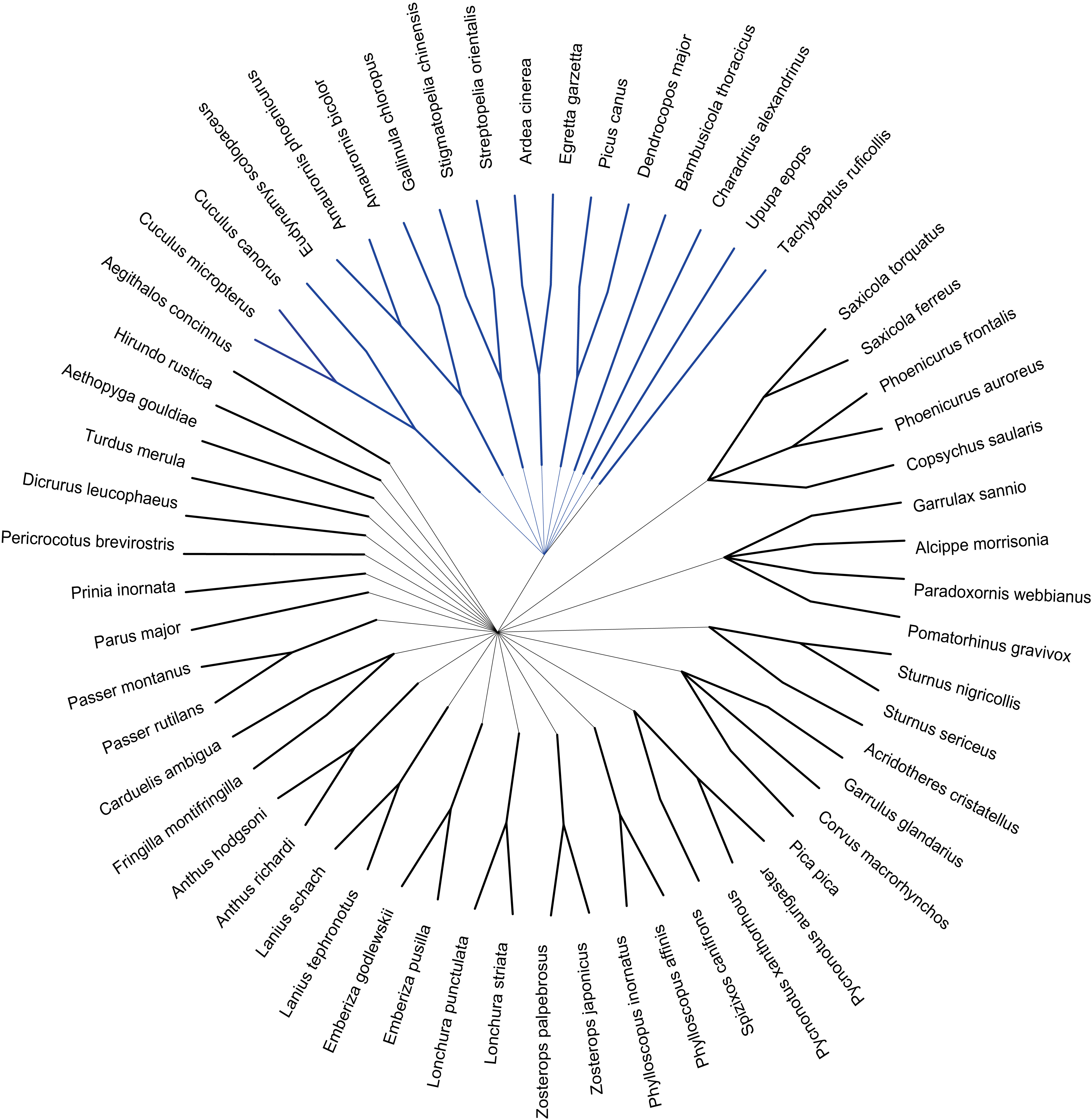

Supplement: Supplementary material 5 — Phylogenetic tree of the 58 bird species in the Xiaohongxiang Wetland [file bdj-12-e136248-s005.jpg]
